# Copy-number-variation and copy-number-alteration region detection by cumulative plots

**DOI:** 10.1186/1471-2105-10-S1-S67

**Published:** 2009-01-30

**Authors:** Wentian Li, Annette Lee, Peter K Gregersen

**Affiliations:** 1 The Robert S Boas Center for Genomics and Human Genetics, Feinstein Institute for Medical Research, North Shore LIJ Health System, Manhasset, NY 11030, USA

## Abstract

**Background:**

Regions with copy number variations (in germline cells) or copy number alteration (in somatic cells) are of great interest for human disease gene mapping and cancer studies. They represent a new type of mutation and are larger-scaled than the single nucleotide polymorphisms. Using genotyping microarray for copy number variation detection has become standard, and there is a need for improving analysis methods.

**Results:**

We apply the cumulative plot to the detection of regions with copy number variation/alteration, on samples taken from a chronic lymphocytic leukemia patient. Two sets of whole-genome genotyping of 317 k single nucleotide polymorphisms, one from the normal cell and another from the cancer cell, are analyzed. We demonstrate the utility of cumulative plot in detecting a 9 Mb (9 ×10^6 ^bases) hemizygous deletion and 1 Mb homozygous deletion on chromosome 13. We also show the possibility to detect smaller copy number variation/alteration regions below the 100 kb range.

**Conclusion:**

As a graphic tool, the cumulative plot is an intuitive and a scale-free (window-less) way for detecting copy number variation/alteration regions, especially when such regions are small.

## Background

Most efforts in genetic mapping of human diseases focus on single-nucleotide-polymorphism (SNP): individual nucleotide base that may differ from one person to another. If the cause of a polymorphism is due to diverging paths in population genetic history, such as in multiple ethnic groups, it can be used as an ancestry or ethnic identity marker [1]. If the polymorphism is a functional mutation (non-synonymous or promoter-region polymorphism) [2] underlying a human disease, then it is the focus of attention in case-control genetic analyses [3].

A new type of genetic polymorphism emerged recently as another source of mutation that may lead to human diseases: the copy number variation (CNV) (for literature on CNV, see an online bibliography [4]). Local duplication and deletion events occuring at kb (10^3 ^bases) or Mb (10^6 ^bases) scales are the cause of CNV. If these events occurred in prior generations, CNV can be treated as a genetic marker whose transmission might be traced in studying the disease-status correlation. These events can also occur in the current generation, as *de novo *mutations.

Similar duplication and deletion events also occur in somatic cells, leading to copy number alteration (CNA). Besides the link between CNA and cancers studied before [5], an early CNV-disease association was reported on Charcot-Marie-Tooth disease [6], in inherited neurological disorder. In the past year or two, the number of reports on association of CNV with human diseases increased dramatically, especially for psychiatric disorders such as Schizophrenia [7-9], bipolar [10], and for brain developmental disorder such as Autism [11-15]. These diseases have long been evading genetic dissection, and the CNV link offers new optimism for our ultimate understanding of these diseases.

The technology for CNV detection evolves from Mb-level comparative genomic hybridization (CGH) to higher-resolution array-based CGH [16]. Genotyping array whose original goal is to genotype individual SNPs, has increasingly been used for CNV detection [17-20]. There are two relevant pieces of information from a genotyping array data for the purpose of CNV detection.

The first is the ratio of intensity reading of alleles for a sample to that from a reference group of normal samples. If the ratio is larger than 1, there are more copies of piece of DNA in the sample than normal (which is 2 copies). If the ratio is less than 1, it indicates a deletion. The second signal is the genotype. Deletion of one of the chromosomes leads to a run of homozygosity for all SNPs in the region, though run of homozygosity can also be due to inbreeding [21,22]. The homozygosity property of one-copy deletion is well exploited in detecting loss-of-heterozygosity in CNA of cancer cells [23].

CNV detection using genotype microarray data relies on these two sequences: if the intensity ratio deviates from the normal value of 1 for a chromosome region with a consistent value, it can be a CNV region. Similarly, if a run of homozygosity is observed in a region, it could indirectly indicate a copy-number deletion. A CNV region detection is more convincing if CNV signals exhibited by both sequences overlap in a common region.

Methods for calling CNV regions can be roughly classified into two types. The first type is straightforward: a CNV detection is claimed when the log-ratio value is significantly deviated from 0 [24]. The problem with this method is that the threshold for calling CNV varies greatly from platform to platform, from study to study, and a comparative investigation is urgently needed [16]. The second type uses hidden Markov models (HMM), where the underlying CNV status is the hidden variable, and the log-ratio and genotype sequences are the two observed variables [25-30]. One advantage of the HMM framework is that it can incorporate information from both sequences at once.

When the parameter settings in a HMM are fixed, HMM is a stationary (homogeneous) process along a chromosome. There is one parameter in HMM which controls the transition probability from the (hidden) CNV state to non-CNV state. That parameter can also be transformed to the characteristic size for CNV region [28]. What if the CNV regions do not have a characteristic size, or equivalently, the length distribution is not exponential? In that case, CNV-calling methods that do not require stationarity are preferred.

The guanine-cytosine content (GC%) in DNA sequences has been a focus of non-stationary, non-Markov, long-range-correlated modeling for more than twenty years [31-33]. It is well acknowledged that the hierarchical pattern of GC%-domains within GC%-domains is possible [34,35]. In order to detect both small and large GC-homogeneous domains, one applies methods that do not preset a characteristic scale. One such method is the recursive segmentation that adopts a divide-and-conquer approach [36]. Another is the cumulative plot.

Cumulative plot is a graphic display of sequence information such that trend in a region becomes more visible and obvious. It is a window-less method because no characteristic scale needs to be specified, although a window can be imposed to a plot when all patterns within certain length scale are to be ignored. In DNA sequence context, such cumulative plots were called "DNA walk" [37,38] or "Z curve" [39,40]. The cumulative plot has also been widely used for detection of replication origin [41,42]. To our knowledge, cumulative plots have not been applied to CNV/CNA detection. The purpose of this paper is not to provide a comprehensive comparison of various CNV/CNA-calling methods, but limited to the presentation and illustration of this new approach.

## Results and discussion

**Figure 1:**
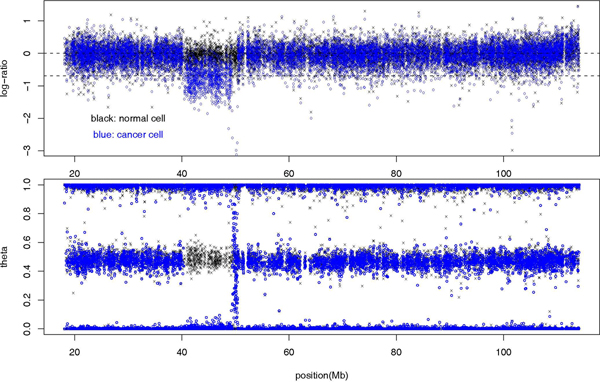
**Log-ratio and genotype sequences for chromosome 13 in paired samples from a CLL patient**. Log-ratio (top) and genotype *θ *(bottom) sequence for SNPs from chromosome 13 of two samples taken from the same cancer patient: black for normal cell and blue for cancer cell. For the log-ratio plot, the copy number of 2 level log(2/2) = 0 and the copy number of 1 level log(1/2) = -0.693147 are marked.

Since our method applies equally to CNV and CNA data, here we examine the CNA pattern in a cancer patient with chronic lymphocytic leukemia (CLL) [43]. DNA samples from the patient's normal cell and that from the cancer cell are obtained and genotyped with 317,000 SNPs genomewide. Figure 1 shows the log-ratio and *θ *sequences (see Methods) for chromosome 13, where a 9 Mb CNA region (deletion) in the cancer cell is clearly visible. A deletion region is characterized by a drop in log-ratio value, and an absence of heterozygosity. Our goal is to capture the same information using cumulative plots.

**Figure 2:**
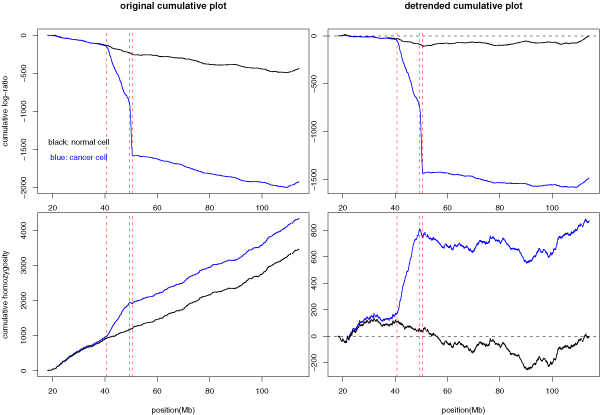
**Cumulative plot of log-ratio and homozygosity sequence**. Cumulative plot and detrended cumulative plot for both the log-ratio sequence and the homozygosity indicator sequence (for the chromosome 13 data shown in Figure 1). Top: cumulative plots for log-ratio sequence. Bottom: cumulative plot for homozygosity sequence (1 for homozygote, -1 for heterozygote). Left: original cumulative plots. Right: detrended cumulative plots. The linear trend obtained from the normal sample is used to detrend both the normal and the cancer sample. Black for the normal cell and blue for the cancer cell. The 9 MB hemizygous deletion and the neighboring 1 Mb homozygous deletion region are marked by red lines.

The left panel of Figure 2 shows the two cumulative plots corresponding to log-ratio sequence and homozygosity indicator sequence *h*, respectively. In the simplest version, at each new SNP, the curve moves up or down by an amount equal to the log-ratio value of that SNP, or by the presence of a homozygote (+1) and a heterozygote (-1).

For a deletion region, the log-ratio value is consistently negative, and the first cumulative plot shows a drop; and genotype is consistently homozygous (also called run of homozygosity (ROH)), and the second cumulative plot shows a jump. However, from Figure 2 (left), even outside the CNA region, the first (second) cumulative plot continues to go down (up), reflecting a global abundancy of negative log-ratio over positive one (homozygotes over heterozygotes).

To remove the global or chromosome-wide average, we redraw a detrended cumulative plot (right panel of Figure 2) where the linear trend from the normal cell is subtracted from the two cumulative plots. If the difference of global trends between the cancer and normal cell is an artifact, e.g., the poor DNA quality in cancer cell that leads to higher missing rate for genotype calling, thus seemingly lower heterozygote frequency, then the normal and cancer cumulative plots should be detrended separately. Without such an evidence, we use the linear trend in normal cell to detrend both samples to highlight the difference between the two.

Cumulative plots can be customized to pick any specially defined signal. Suppose we are mainly interested in regions with copy number equal to 1, i.e., hemizygous deletion. Such deletion region should exhibit two features: (1) log-ratio is equal to log(1/2) = -0.693147 (as versus log(2/2) = 0 in the normal situation); (2) homozygosity indicator equal to 1 (as versus to a mixture of -1's and 1's). For a SNP, we then define a "one deletion" indicator variable whose value is 1 if -2 < log-ratio < -0.34657 (mid-point between -0.693147 and 0) and if its genotype is a homozygote and the value is -1 otherwise.

**Figure 3:**
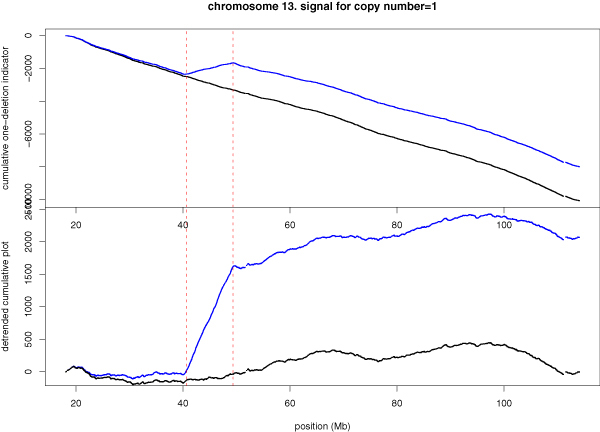
**Cumulative plot of the hemizygous deletion indicator variable**. Cumulative plot (top) and detrended cumulative plot (bottom) for the 9 Mb hemizygous deletion region on chromosome 13, using the "one deletion" indicator variable.

Figure 3 shows the cumulative plot for "one deletion" indicator variable, without or with detrending (by the linear trend in the normal sample). In both versions, the hemizygous deletion region can be seen clearly. Not only the cumulative plot detects the CNA region easily, but also it delineates the border of the deletion region accurately.

When deletion occurs in both chromosomes, called homozygous deletion, the copy number is equal to zero. For homozygous deletions, both A- and B-channel intensity (see Methods) is close to zero, and the log(*r*) is a large negative value. Because in the A- and B-channel plane (see Methods), these SNPs are near the origin, the angle *θ *can not be determined unambiguously. This leads to a broad distribution of *θ *values between 0 and 1, as can be seen from Figure 1 (top).

**Figure 4:**
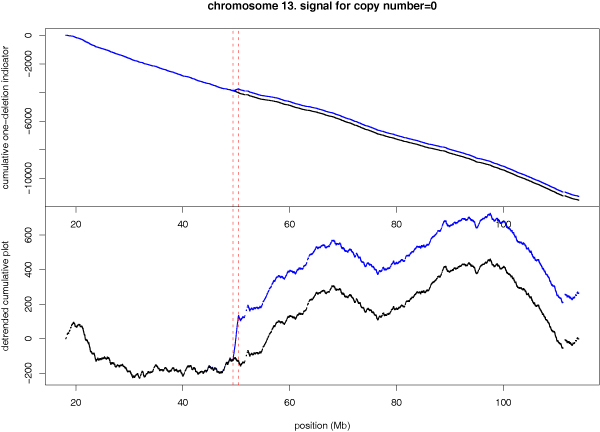
**Cumulative plot of the homozygous deletion indicator variable**. Cumulative plot (top) and detrended cumulative plot (bottom) for the 1 Mb homozygous deletion region on chromosome 13, using the "two deletions" indicator variable.

We define a "two deletions" indicator variable whose value is 1 if the log-ratio is < -2; and the value is -1 otherwise. Note that the genotype information is not used. Figure 4 shows the cumulative plot for the "two deletions" indicator variable for chromosome 13. One homozygous deletion region with ~1 Mb is clearly identified immediately adjacent to the 9 Mb hemizygous deletion region.

The 9 Mb deletion on chromosome 13 in our CLL sample, which was one of the known common deletions for this disease [44], represents an example of easy detection of CNA/CNV region, because the difference between the normal and cancer cell for both log-ratio and genotype sequence is already obvious from the raw data (Figure 1). The advantage of cumulative plot is perhaps its ability to detect CNA/CNV region of smaller sizes.

**Figure 5:**
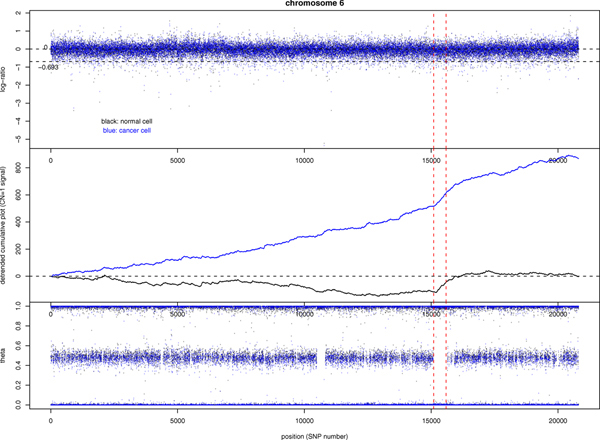
**Log-ratio and genotype sequences for chromosome 6 in paired samples from a CLL patient**. The log-ratio sequence (top), genotype *θ *sequence (bottom), and the detrended cumulative plot for the "one deletion" indicator variable for SNPs on chromosome 6. Black and blue color refer to the normal and cancer cell sample taken from the same cancer patient. The largest run-of-homozygosity region is marked by red vertical lines. The copy number of 2 level log(2/2) = 0 and the copy number of 1 level log(1/2) = -0.693147 are marked in the log-ratio plot.

**Figure 6:**
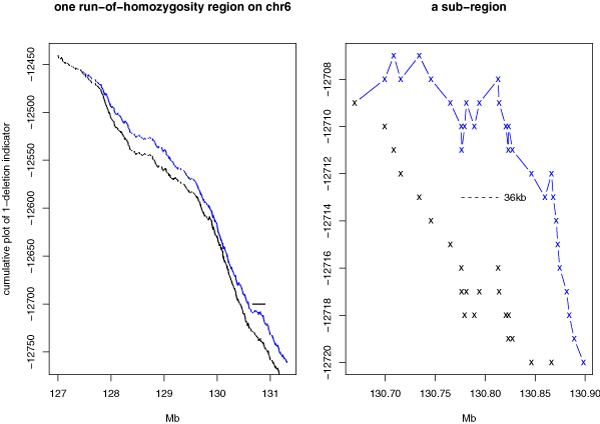
**Zoom in of smaller regions**. Cumulative plots of "one deletion" indicator variable for the region marked in Figure 5 (left), and the sub-region marked by a horizontal bar on the left (right). Black and blue refer to the normal and the cancer sample.

Figure 5 shows the example of chromosome 6 of our sample where there is no large-scaled CNA region. The log-ratio and genotype sequence look almost identical between the normal and the cancer cell. The cumulative plot for the "one deletion" indicator variable shows that there are +400 more SNPs in the cancer cell than in the normal cell to have the one deletion signal (the "two deletion" cumulative plot is not shown because the signal is mostly absent along the chromosome). However, these SNPs are distributed throughout the chromosome, instead of forming clusters, and we still do not have strong evidence that the cancer sample has more micro deletion regions as compared to the normal sample. In order to explore the possible existence of smaller CNA regions, we pick the longest ROH region (roughly 4 Mb) and view it with cumulative plots. Figure 6 (left) shows the un-detrended cumulative plot for the one-deletion indicator variable in this region. A clear hemizygous deletion region should show up as a jump in the cumulative plot. However, the tendency within this ROH region is downward instead of upward. In other words, although all genotypes in this region are homozygous, the log-ratio mostly fails the <-0.34657 criterion.

The failure in detecting hemizygous deletion at the Mb scale does not necessarily prevent its possible existence at a smaller length scale. The right panel of Figure 6 shows a 200 kb sub-region (marked in Figure 6 (left)) that contains a 36 kb region with an upward trend in the cumulative plot. A zooming into any small region in a cumulative plot enables it to detect CNA/CNV regions with ever smaller sizes.

It was previously suggested that run of homozygosity can be a sequence feature that is associated with certain human diseases [45]. We see here that ROH is only a partial indicator for a CNA/CNV region. The longest ROH on chromosome 6 in our sample only shows some weak evidence in a much narrower region for one-deletion CNA. Considering both ROH and log-ratio sequence is clearly better than considering ROH alone. Although ROH may still be biologically meaningful, as it could reflect a copy-neutral loss-of-heterozygosity event, one has to obtain extra evidence to exclude population genetics events such as inbreeding as the true cause.

The pairing of the normal and the cancer sample is not essential to our method. In Figures 3, 4, 6, the CNA regions can be identified by cancer sample (the blue curve) alone. However, the comparison with the normal sample provides supporting evidence that deletion only occurs in the cancer cell and not in the normal cell. When SNPs along a chromosome are not evenly distributed, it may not be appropriate to move one step per SNP in the cumulative plot. For example, if multiple SNPs are in strong linkage disequilibrium in a densely typed region, the indicator variable values are positively correlated, and a sequence of +1 values is partially a consequence of their correlation, not as a series of independent evidences for CNA/CNV. We can adjust for this correlation by calculating the probability ratio *α *(see Method) in favor for concordant genotypes between neighboring SNPs, as compared to the average. If *α *> 1, we discount a +1 (or -1) movement by dividing the *α *value. For the chromosome 13 data, a is in a very narrow range of (0.9921, 1.0002). Because the probability ratio in favor of concordant homozygotes is so close to 1, the adjusted cumulative plot is indistinguishable from the original cumulative plot.

So far the delineation of an upward trend in the cumulative plot is determined by visual inspection. Segmentation programs can be developed to carry out the delineation automatically. In particular, one may move along the cumulative plot, calculate the slope from the start point to the moving position, then from the moving position to the end point. The position that maximizes the difference of the two slopes is chosen, leading to the first segmentation. This segmentation can be carried out recursively similar to the method described in [36].

Finally, for case-control analysis using CNV, one deals with two groups of samples [46]. In this situation, cumulative plot can be first applied to each individual person to identify the CNV/CNA region. Then, chromosomes can be partitioned into equal-sized windows and the frequency of CNV/CNA-containing window in the case group is compared to that in the control group for a statistical test.

## Conclusion

We have shown here that cumulative plots of an indicator variable derived from the log-ratio and SNP genotype sequence can easily identify CNV or CNA regions. We illustrate the procedure for hemizygous deletion (copy number equal to 1) and homozygous deletion (copy number equal to 0) using samples taken from a chronic lymphocytic leukemia patient. Although CNV/CNA regions at the Mb scale can also be detected by viewing the raw data, cumulative plot is able to delineate the borders with higher degree of accuracy. Another advantage of cumulative plot is perhaps in detecting smaller CNV/CNA regions, such as those in the range of 10 kb–100 kb, as it is a scale-free approach that does not require a fixing of the window size. Cumulative plot is simple enough that no special-purpose program is needed for its use except a graphic routine: for example, all results shown here are obtained by the general statistical package R [47].

## Methods

### log-ratio and genotype data

In a two-channel (two-color) SNP genotyping microarray, the A- and B- channel (A- and B-allele) intensity reading is recorded. These two intensities are normalized by reference intensity values which are obtained by averaging many normal samples. Each SNP can be represented by a point in the (*x*, *y*) plane where *x*, *y *are the normalized A- and B-channel intensity. The polar coordinate of the point is $r=\sqrt{x^{2}+y^{2}}$ and *θ *= *tan*^-1^(*y*/*x*) [48]. Log(r) is the "log ratio" value that provides a copy-number information, and *θ *provides a genotype information, where *θ *= 0, 1 correspond to two homozygotes, and *θ *= 0.5 corresponds to the heterozygote. Note: (1) *r *value depends on a group-averaged reference level, and this information is provided by the array-maker company. (2) Although *r *and *θ *is in principle independent, there could be weak correlation between them. Our starting point are the two sequences of log(r) and discretized *θ *values (i.e. genotype) along a chromosome.

### Cumulative plots for log-ratio and homozygosity sequence

The *r *and *θ *variable is transformed by: log-ratio = *log*(*r*) and homozygosity indicator *h *= 4 × |*θ *- 0.5| - 1. For heterozygotes, *h *is close to -1, and for two homozygotes, *h *is close to 1. Denote the *i*-th SNP's log-ratio and homozygosity indicator as log(*r*_*i*_) and *h*_*i*_. The (original) cumulative plots of these two sequences are:

(1) $$\begin{array}{c} cumu.log.ratio_{j}=\sum_{i=1}^{j}\log(r_{i})\\ cumu.h_{j}=\sum_{i=1}^{j}\left(h_{i}\right) \end{array}$$

A cumulative plot can be detrended such that the first and the last SNP are on the same horizontal line. The purpose of this detrending is to remove the chromosome-wide bias so that regional deviations are highlighted. In our normal and cancer cell from the same individual example, we detrend the normal sample by subtracting the linear function a + *bx*_*i*_, where *x*_*i *_is the Mb position of the *i*th SNP, *N *is the number of SNPs, and

(2) $$\begin{array}{c} b=\frac{cumu.log.ratio_{N}-cumu.log.ratio_{1}}{x_{N}-x_{1}}\\ a=cumu.log.ratio_{N}-bx_{N}. \end{array}$$

To highlight the difference between the cancer cell and the normal cell, we use the *a *and *b *obtained from the normal cell to detrend the cumulative plot for the cancer cell.

### Cumulative plots corrected by spacing between neighboring SNPs

When SNPs are not distributed evenly along a chromosome, one may consider correcting the effect of inhomogeneous correlation between neighboring SNPs. We first calculate the probability of a neighboring SNP of a homozygous SNP to be also homozygous due to the correlation between them. This calculation is carried out by the Haldane's map [49].

Haldane's map relates the number of recombinations within a chromosomal interval *M *and the probability of observing a recombinant between the two end points *R*:

(3) $$R=\frac{1-exp(-2M)}{2}.$$

The unit of *M *is Morgan, which is roughly equal to 100 Mb (or 1 centi Morgan is equal to 1 Mb [50]). The probability of observing a non-recombinant is 1 - *R*.

Denote *p*_*same *_the probability that one homozygous SNP is followed by another homozygous SNP that is *M *genetic distance apart. Since Haldane formula is applicable to haplotype, or a single copy of a chromosome, for two copies of a chromosome, *p*_*same *_= (1 - *R*)^2 ^≈ 1 - 2*R *= *e*^-2*M*^.

Suppose the average spacing between two neighboring SNPs is $\bar{M}$. For a neighboring SNP pair whose spacing M <$\bar{M}$, it is more likely for both SNPs to be homozygous than the average, by a probability ratio of $\alpha =p_{same}/{\bar{p}}_{same}=e^{-2(M-\bar{M})}$, and the cumulative plot for the homozygosity indicator variable can be adjusted by dividing that ratio:

(4) $$cumu.h_{j}=\sum_{i=1}^{j}h_{i}/\alpha_{i}=\sum_{i=1}^{j}h_{i}e^{2(M_{i-1,i}-\bar{M})}.$$

We assume that *p*_*same *_is calculated in the same way as for other indicator variables, meaning CNV/CNA of a particular type is maintained at the neighboring SNP by the same probability *e*^-2*M*^, and the above formula can be used to correct other cumulative plots. Note that transition probability from one genotype in a SNP to another genotype in the neighboring SNP can also be estimated from the HapMap data.

## List of abbreviations used

CGH: comparative genomic hybridization; CLL: chronic lymphocytic leukemia; CNA: copy number alterations; CNV: copy number variations; GC%: guanine and cytosine contents; HMM: hidden Markov models; ROH: run of homozygosity; SNP: single nucleotide polymorphism

## Competing interests

The authors declare that they have no competing interests.

## Authors' contributions

W.L. designed the method, carried out the analysis, and wrote the manuscript; A.L. genotyped the samples; P.K.G. proposed the CNV study of chronic lymphocytic leukemia.
